# Physiological and Pathological Ageing of Astrocytes in the Human Brain

**DOI:** 10.1007/s11064-021-03256-7

**Published:** 2021-02-08

**Authors:** Marloes Verkerke, Elly M. Hol, Jinte Middeldorp

**Affiliations:** 1 Department of Translational Neuroscience, University Medical Center Utrecht Brain Center, Utrecht University, Utrecht, The Netherlands; 2 Department of Immunobiology, Biomedical Primate Research Centre (BPRC), P.O. Box 3306, 2280 GH Rijswijk, The Netherlands

**Keywords:** Human astrocyte, Ageing, APOE, Alzheimer’s disease, iPSC, Post-mortem human brain tissue, Reactive gliosis

## Abstract

Ageing is the greatest risk factor for dementia, although physiological ageing by itself does not lead to cognitive decline. In addition to ageing, *APOE ε4* is genetically the strongest risk factor for Alzheimer’s disease and is highly expressed in astrocytes. There are indications that human astrocytes change with age and upon expression of APOE4. As these glial cells maintain water and ion homeostasis in the brain and regulate neuronal transmission, it is likely that age- and APOE4-related changes in astrocytes have a major impact on brain functioning and play a role in age-related diseases. In this review, we will discuss the molecular and morphological changes of human astrocytes in ageing and the contribution of APOE4. We conclude this review with a discussion on technical issues, innovations, and future perspectives on how to gain more knowledge on astrocytes in the human ageing brain.

## Introduction

Ageing affects every part of the human body differently, with some showing evident signs of this process like the skin undergoing atrophy [1]. On the contrary, the brain is fairly resilient to this process of decay, as physiological ageing does not lead to major neuronal and glial cell death or severe cognitive impairment [2–4]. Nonetheless, ageing is the most important risk factor for neurodegenerative diseases such as Alzheimer’s Disease (AD). This suggests that more subtle changes occur with advancing age, which makes the brain more vulnerable to age-related diseases, i.e. pathological ageing. In this review, we focus on age-related changes in human astrocytes and implications for astrocyte function.

**Fig. 1:**
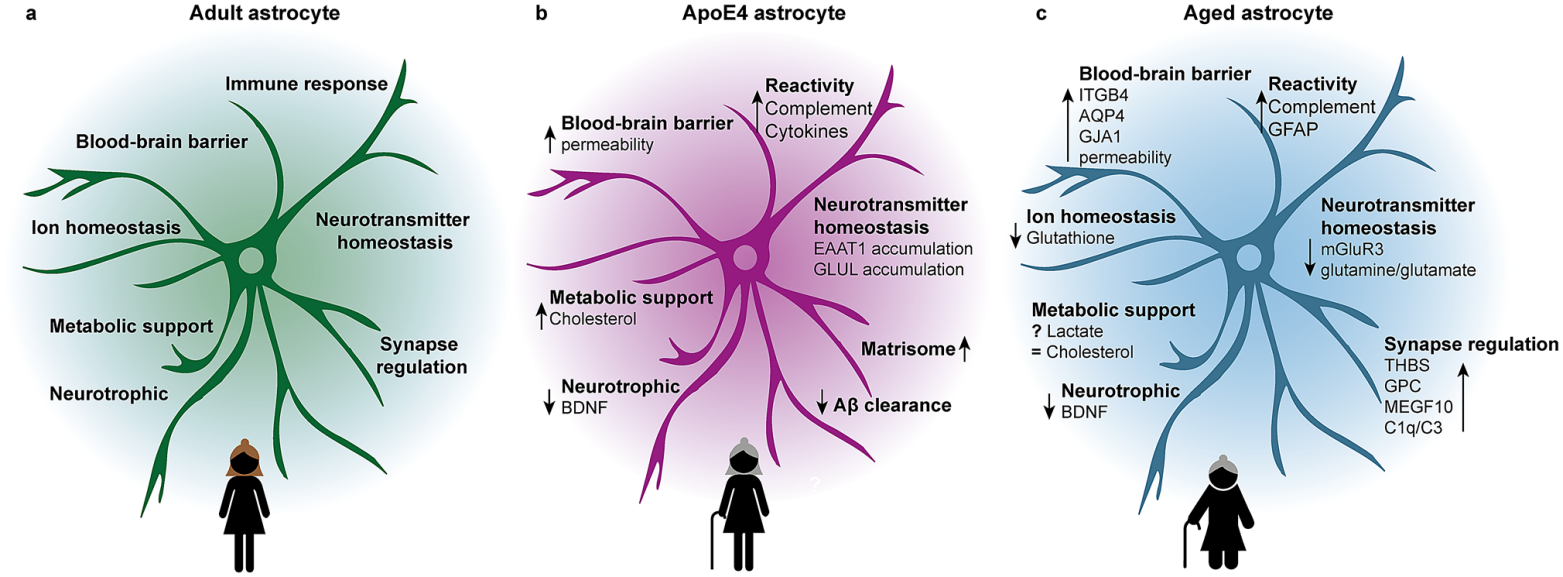
Ageing of astrocytes in the human brain. **a** In the adult human brain, astrocytes are part of the blood–brain barrier (BBB), maintain ion homeostasis, support and regulate neuronal transmission, and are involved in immune response of the brain. **b** Human *APOE ε4*/*ε4* astrocytes in vitro display an aged molecular profile. BBB permeability is increased and cholesterol metabolism is altered. There is less secretion of neurotrophic BDNF and neurotransmitter homeostasis is affected by accumulation of EAAT1 and GLUL. Complement and cytokine secretion indicate an inflammatory profile. These changes are overlapping with aged astrocytes. In addition, *APOE ε4*/*ε4* astrocytes increase the expression of matrisome-related molecules and are less capable of clearing Aβ. This early ageing phenotype possibly underlies the increased risk of developing AD in *ε4* carriers. **c** Ageing of the human brain induces molecular changes in astrocytes. Molecules involved in the BBB are upregulated (ITGB4, AQP4, GJA1) and BBB permeability increases. The release of the antioxidant glutathione is reduced in aged astrocytes, leading to a decreased protection against ROS. Metabolic support does not seem to change with ageing. Regarding neuronal transmission, aged astrocytes secrete less neurotrophic BDNF and are less efficient in maintaining neurotransmitter homeostasis due to reduction of mGluR3 expression and decreased conversion of glutamate into glutamine. Molecules involved in synaptogenesis and pruning are all upregulated (THBS, GPC, MEGF10, C1q, C3). Also, aged astrocytes display an inflammatory profile indicated by increased GFAP and complement levels. *ITGB4* integrin beta 4; *AQP4* aquaporin-4; *GJA1* connexin 43; *ROS* reactive oxygen species; *BDNF* brain-derived neurotrophic factor; *THBS* thrombospondins; *GPC* glypicans; *MEGF10* multiple epidermal growth factor-like domains protein 10; *GFAP* glial fibrillary acidic protein; *EAAT1* excitatory amino acid transporter 1; *GLUL* glutamine synthetase; *Aβ* amyloid beta; *AD* Alzheimer’s disease; ? means contradictory results; – means not changed

Astrocytes are a major glial cell type in the human brain, harbouring homeostatic and neuromodulating functions (Fig. 1a) [5]. These cells maintain ion homeostasis by regulating water transport and potassium, calcium, and chloride levels. In addition they maintain homeostasis of reactive oxygen species (ROS) by releasing antioxidants like glutathione, which is important in the context of ageing [6]. Depending on the demand of neurons, astrocytes provide metabolic substrates, such as glycogen and lactate [7]. Moreover, they maintain not only neurotransmitter homeostasis via transporters like glutamate and GABA transporters, but also actively shape neuronal networks through regulation of synapse formation and pruning, together with microglia [8–11]. With their endfeet, they form part of the blood–brain barrier (BBB) and the brain-cerebrospinal fluid (CSF) barrier in the pial layer [6, 12]. Finally, astrocytes are, together with microglia, responsible for the immune response of the brain upon an infection, a trauma, or brain disease [6]. Given this extensive set of functions performed by astrocytes, we expect that age-related changes in astrocytes have a major impact on brain functioning.

Besides this array of functions, human astrocytes are heterogenous in morphology and transcriptional profile. In the adult human cortex, four types of astrocytes have been described based on morphology: interlaminar astrocytes, protoplasmic astrocytes, varicose projection astrocytes, and fibrous astrocytes [5]. These subtypes are distinguished based on the glial fibrillary acidic protein (GFAP), which is the main intermediate filament (IF) protein in astrocytes [13]. It should be kept in mind that immunolabeling of GFAP only reveals the filamentous skeleton of an astrocyte and therefore does not reflect the full morphology. The fine processes that make up most of the astrocytes cannot be visualized with GFAP immunostaining. Furthermore, GFAP shows transcriptional variation as some astrocytes express low levels of GFAP, while others have a higher baseline GFAP expression [14–16]. Likewise, other astrocyte markers present regional variation. For example, cortical astrocytes express relative high levels of ALDH1L1 and low levels of GFAP [17, 18]. Still, GFAP is often used as the golden standard to identify astrocytes, but region-specific expression patterns indicate that a proportion of astrocytes is missed if only GFAP as a marker is used. This morphological and transcriptional heterogeneity should be considered when studying astrocytes in the human brain [14, 19]. Thus, identifying astrocytes solely based on GFAP expression will bias studies towards a specific subpopulation and this is a challenge that needs to be faced to enhance our understanding of astrocytes.

During physiological ageing, the brain changes including its astrocytes. As cell numbers do not change, subtle alterations must underlie the increased vulnerability for pathological ageing processes such as AD. On the contrary, in AD there are unquestionable changes in cell numbers due to degeneration which leads to severe cognitive decline and these pathological processes cannot be ascribed to physiological ageing processes [2]. Thus, another stimulus will likely tip the balance from physiological to pathological ageing. A possible candidate is apolipoprotein E4 (APOE4), the largest genetic risk factor for AD [20, 21]. This protein is highly expressed by astrocytes and therefore it is plausible that astrocytic changes underly the increased risk of AD [22]. In this review, we will describe transcriptional and IF-related morphological changes of human astrocytes during physiological ageing and induced by APOE4. We will compare these changes to pathological ageing in AD and hypothesize how these are interconnected.

## Molecular Profile of Human Astrocytes in Physiological Ageing

In the last decade, gene expression profiling studies (microarray and RNA-sequencing) provided detailed insight in cell-type-specific transcriptomic changes across different brain regions in the ageing human brain [14, 23–27]. When analysing gene expression in various neural cell populations from different brain regions and different ages, changes in neuron-specific gene expression cluster per brain region [27–29]. Instead, changes in glia-specific gene expression, among which astrocyte genes, show a stronger correlation with age [25–27, 30].

To date, only a few transcriptomic studies have described astrocyte-specific gene expression in the human brain (Table 1). Two studies analysed the transcriptome of individual cell types, one in laser-captured GFAP positive astrocytes [30], the other in several astrocyte subpopulations identified by single nuclear RNAseq [26]. Both studies are focussed on AD pathology and only have data from elderly (> 71 years old) [26, 30], thus lacking a comparison to younger individuals. Therefore, it is hard to say anything about age-related changes in these studies. Habib et al. (2020) used the human dataset from Mathys et al. (2019) to compare with their mouse RNAseq data and identified a continuous range of astrocyte subpopulations categorized by *GFAP* expression [14]. The other four transcriptomic studies, Kumar et al. (2013) [23], Simon et al. (2018) [22, 31, 32], Soreq et al. (2019) [33–35], and Wruck et al. (2020) [33, 36–41], made use of bulk brain tissue transcriptomics. Although an astrocyte-specific human ageing study has not yet been performed, for this review we extracted astrocyte-specific changes from these eight studies.

**Table 1:** Transcriptional studies into human ageing

Publication	Experimental set-up		Age range in years	M/F	Brain region
Simpson et al. (2011) [30]	Microarray	Laser captured GFAP positive astrocytes	71–103	5/13	TCX
Kumar et al. (2013) [23]	Microarray	Bulk tissue	15–91	223/96	CBL/FCX
Simon et al. (2018) [42]	Meta-analysis	RNAseq – bulk [31]	78–100	25/29	HIPP/PCX/TCX/WM
		RNAseq—bulk [22]	8–65	22	FCX/HIPP/TCX
		Microarray – bulk [32]	84 ± 11	37/42	FCX/HIPP/TCX
Payan-Gomez et al. (2018) [43]	Meta-analysis	Microarray – bulk [38]	24–29 71–95	10/9	PFC
		Microarray – bulk [44]	0–47.4	26/13	PFC
		Microarray – bulk [45]	28–97	13/10	PFC
		Microarray – bulk [46]	0–98	18/5	PFC
		Microarray – bulk [39]	16–96	332/88	PFC
Mathys et al. (2019) [26]	RNAseq	Single nuclei	75–90+	24/24	PFC
Soreq et al. (2019) [27]	Meta-analysis	Microarray – bulk [35]	16–102	134	CBL/FCX/HIPP/ MED/OCX/PUT/SN/ TCX/THA/WM
		Microarray – bulk [34]	16–101	204/101	CBL – FCX
		Microarray – bulk [33]	24–106	39/0	FCX
		RNAseq – bulk [22]	8–65	22	FCX/HIPP/TCX
Habib et al. (2020) [14]	RNAseq	Single nuclei – mouse data compared to [26]	75–90+	24/24	PFC
Wruck et al. (2020) [25]	Meta-analysis	Microarray – bulk [36]	18–81	24/5	PFC
		Microarray – bulk [37]	25–94	11/8	PFC
		Microarray – bulk [33]	24–106	20/21	PFC
		Microarray – bulk [38]	24–29 71–95	10/9	PFC
		Microarray – bulk [39]	16–96	332/88	PFC
		Microarray – bulk [40]	52 ± 15	32/20	PFC
		RNAseq – bulk [41]	–	8/8	PFC

Symbols and abbreviations: *CBL* cerebellum, *FCX* frontal cortex, *HIPP* hippocampus, *MED* medulla, *OCX* occipital cortex, *PCX* parietal cortex, *PFC* prefrontal cortex, *PUT* putamen, *SN* substantia nigra, *TCX* temporal cortex, *THA* thalamus, *WM* white matter

Interestingly, of the 10 brain regions studied by Soreq et al. (2017) this age-related loss of region-specificity is most pronounced in the hippocampus and substantia nigra, two key areas in age-related neurodegenerative diseases such as AD and Parkinson’s disease (PD) [27]. Gene ontology (GO) and pathway analyses of differentially expressed genes (DEGs) can give insight into broader functional changes that are related to the transcriptional changes. Astrocyte-specific DEGs upregulated with age were enriched in pathways such as “inflammation”, “cell–cell adhesion”, “cell morphogenesis”, and “extracellular matrix organization” [25, 27]. A decreased expression with age is only shown by a few astrocyte-specific genes, which are related to the glutamate system in pathways such as “glutamate receptor signalling” and “synaptic transmission, glutamatergic” [25]. These pathways hint towards altered homeostatic functions in human aged astrocytes. Next, we will discuss these groups of astrocyte functions that changed based on human transcriptional studies. Besides, we will discuss whether astrocyte functions that are changed in rodent ageing studies, are also represented in human (transcriptional) studies.

### Altered Homeostatic Functions of Aged Human Astrocytes

Astrocytes are involved in molecular, metabolic, and cellular brain homeostasis [6]. We will describe age-related changes divided into three categories of homeostatic functions, namely neuronal support functions, regulation of synapse formation and function, and maintaining the BBB (Fig. 1c).

#### Neuronal Support Functions

One of the homeostatic functions that astrocytes exhibit is the protection of neurons against free radicals and oxidative stress. The oxidative stress theory of ageing states that accumulation of ROS-induced damage underlie age-related changes [47–49]. Astrocytes release glutathione, which is an endogenous antioxidant that protects neurons from free radicals such as ROS [50]. In the aged human brain, glutathione levels are reduced in astrocytes thereby hampering this homeostatic function [51, 52]. Another homeostatic function of astrocytes is providing metabolites to neurons. Lactate is produced by astrocytes via glycolysis and when secreted it can be used as an energy source by neurons [7]. Studies on lactate levels in the aged human brain have been inconsistent, reporting either an increase or a decrease with advancing age [7, 51, 53]. Astrocytes also provide neurons with cholesterol to maintain membrane integrity [54]. Whereas rodent studies show a reduction in cholesterol synthesis [25, 27, 55], no human studies are known to indicate that this process is affected by ageing in astrocytes. To conclude, homeostatic functions regarding neuronal support seem not to be majorly changed in aged human astrocytes.

#### Regulation of Synapse Formation and Function

Astrocytes have thin protrusions that intimately interact with synapses, forming the tripartite synapse [56]. These perisynaptic processes express proteins that are involved in synaptic transmission, among which metabotropic glutamate receptors (mGluRs), glutamate transporters, and glutamine synthetase (GLUL), which bind, transport, and convert glutamate, respectively. MGluR3 and mGluR5 are the most abundant mGluRs on astrocytes in the human brain [57, 58]. They are activated by glutamate overspill from the synapse, resulting in local calcium concentration increase and subsequent release of gliotransmitters [57]. The mGluR3 shows a decrease with advancing age [25, 59] and the enzyme GLUL seems less efficient in the aged human brain as demonstrated by an increased glutamine/glutamate ratio [60]. Astrocytes also secrete neurotrophic molecules to support neurogenesis and synaptogenesis. For example, brain-derived neurotrophic factor (BDNF), involved in synaptic plasticity, is decreased in the ageing human brain [61]. Such changes in neurotropic function and glutamate homeostasis might hamper the capacity of astrocytes to regulate synaptic processes in the aged human brain.

In the developing and healthy adult brain, astrocytes play a role in synapse formation by expressing molecules such as thrombospondins, SPARC, Hevin, and glypicans to regulate cell–cell interaction [10, 11, 62]. Subsequently, fine-tuning of synaptic transmission is done by synaptic pruning, a task mainly performed by microglia through complement-mediated synapse removal involving C1q and C3 [9], but astrocytes can also engulf synapses through phagocytosis via MERTK and MEGF10 [8]. In human astrocytes, glypicans, thrombospondins, and MEGF10 are upregulated with age in many brain regions, except in the frontal cortex and hippocampus where MEGF10 is downregulated [27, 63]. Furthermore, both *C1q* and *C3* are upregulated in the aged human brain, which overall hints towards increased synaptic pruning in the aged human brain [64, 65].

#### Blood–Brain-Barrier Regulation

Together with endothelial cells and pericytes, astrocytes are part of the BBB, which limits the influx of blood components, pumps out cerebral waste materials, and regulates the movement of amino acids and glucose into the cerebral parenchyma to support the function and survival of brain cells. One of the molecules in astrocytes that is essential for the formation of the BBB is integrin β4 (ITGB4). This gene, which is involved in cell–cell adhesion and extracellular matrix (ECM) interaction [22, 66], is among the 10 genes most significantly correlated with age in the human prefrontal cortex [25]. Since in rodent studies an upregulation of *Itgb4* is linked to reactive astrocytes [67, 68], this could be indicative of an inflammatory profile of these aged human astrocytes.

Because astrocytes have terminal processes on both synapses and the brain vasculature, they can modulate neuronal activity and cerebral blood flow, so-called ‘neurovascular coupling’ [69]. Moreover, the vascular endfeet, which are interconnected by gap junctions through connexin 43 and 30 (GJA1; *GJB6*), express the potassium channel Kir4.1 and water channel aquaporin-4 (AQP4) to regulate ion concentrations and water balance [5]. Transcriptomic data shows that both *AQP4* and *GJA1* are upregulated with ageing in the frontal, occipital, and temporal cortices, hippocampus, putamen, medulla, white matter, and cerebellum [14, 27]. This upregulation of *AQP4* is confirmed on protein level in the frontal and occipital cortex [70, 71]. In a broader perspective, BBB integrity, measured by magnetic resonance imaging (MRI), shows no significant changes with increasing age in the frontal and temporal cortex, however, in the hippocampus there is an age-dependent progressive loss of BBB integrity [72]. This is consistent with the meta-analysis of Farrall and Wardlaw (2009) that shows an increased permeability with ageing [73]. If and how human astrocytes are implicated in this loss of BBB integrity is to date not known.

In conclusion, there are multiple indications that astrocyte homeostatic functions are altered in the aged human brain, which is in corroboration with rodent studies [74–76]. These homeostatic changes could go hand in hand with an increase in the inflammatory function of astrocytes, which is discussed below.

### Inflammatory Profile of Aged Human Astrocytes

Neuroinflammation is associated with the ageing brain and both astrocytes and microglia are involved [77–79]. Reactive astrogliosis is a process in which astrocytes activate molecularly defined programs resulting in biochemical, morphological, metabolic, and transcriptional changes, which leads to a gain of new functions or alterations in homeostatic functions [15, 80, 81]. One gene that is significantly upregulated in reactive astrocytes is GFAP [15]. Immunostainings with antibodies against this IF protein can visualize the hypertrophic morphology typical for reactive astrocytes, and therefore GFAP is often used as a marker for astrocyte reactivity [15, 80]. Expression of GFAP in the brain has been studied across human lifespan in various brain regions (Table 2). It is consistently seen that the expression of GFAP, both on mRNA as well as protein level, increases with age, especially around the age of 70 [82, 83]. This increase is most prominent in the hippocampus, but also present in frontal, temporal, and entorhinal cortices, and substantia nigra [82–85]. Based on the GFAP immunoreactivity pattern, the astrocyte IF network changes morphologically from long, thin processes in younger individuals, to short, stubby processes in older individuals (Table 2) [85, 86]. While this resembles the hypertrophic morphology of reactive astrocytes [80, 87], systematic human studies into this morphological change with various methods across different brain regions are limited. Both the upregulation of GFAP and the morphological changes would endorse the “inflammageing” theory, which states that ageing induces a chronic, low-grade inflammatory status [88]. However studies should not only rely on an increase in GFAP in the human ageing brain to prove a reactive astrocyte phenotype [80, 89].

**Table 2:** GFAP in the ageing human brain

Publication	Experimental set-up		Age years	N	Brain region	Expression		Morphology
Nichols et al. (1993) [82]	RNA	RNA blot	25–79	47	HIPP FCX TCX	↑ ↑ ↑	≥ 60 years old	–
David et al. (1997) [83]	Protein	WB	12–98	33	HIPP ECX FCX PCX TCX	↑ ↑/~ ↑ ↑	≥ 65 years old	–
Cruz-Sanchez et al. (1998) [86]	Protein	IHC	21–96	40	CX SPC	~ ↑	≥ 75 years old	~ Decrease in thin processes
Del Valle et al. (2003) [84]	Protein	IHC	30–44 82–88	20	CX	↑	82–88 years old	–
Jyothi et al. (2015) [85]	Protein	IHC	0–88	36	SN	↑	Correlated with age	Short, stubby instead of long slender processes
Wruck et al. (2020) [25]	mRNA	Microarray	< 35 35–65 65 >	591	PFC	↑	Correlated with age	–

Symbols and abbreviations: *CX* cortex, *ECX* entorhinal cortex, *FCX* frontal cortex, *HIPP* hippocampus, *PCX* parietal cortex, *PFC* prefrontal cortex, *SPC* spinal cord, *SN* substantia nigra, *TCX* temporal cortex, – not mentioned, ↓ decrease, ↑ increase, ~ not changed, *IHC* immunohistochemistry, *WB* Western blot, *GFAP* glial fibrillary acidic protein

#### Indications from Mouse Ageing Studies

In search for age-related reactive astrocyte markers, we reviewed several hallmark papers on transcriptomic changes in mouse astrocytes in response to CNS injuries and ageing (Table 3).

**Table 3:** Transcriptional studies into murine astrocyte ageing

Publication	Experimental set-up		Age	Sex	Brain region
Orre et al. (2014) [90]	Microarray	FACS GLT1 + /CD11b-	2.5 months 15–18 months	Male Female	CX
Clarke et al. (2018) [94]	RNAseq	TRAP Aldh1l1-eGFP-L10a mice	P7, P30, 10 weeks, 9.5 months, 2 years	Male Female	CX HIPP STR
Boisvert et al. (2018) [55]	RNAseq	Ribotag Floxed-Rpl22-HA x GFAP-cre mice	4 months 2 years	Male	VCX MCX HYP CBL
Pan et al. (2020) [93]	RNAseq	FACS ACSA2+	2, 4, 6, 9, 12 months	Male	WB

Symbols and abbreviations: *CBL* cerebellum, *CX* cortex, *HIPP* hippocampus, *HYP* hypothalamus, *MCX* motor cortex, *STR* striatum, *VCX* visual cortex,– not mentioned, *GFAP* glial fibrillary acidic protein, *GLT1* glutamate transporter 1, *ACSA2* astrocyte cell surface antigen-2, *ALDH1L1* aldehyde dehydrogenase 1 L1

We were the first to analyse DEGs between young adult and aged mouse astrocytes and showed that genes with increased expression in cortical aged astrocytes were implicated in biological processes such as ‘defence response’ and ‘antigen presentation’ [90]. These processes were also upregulated in astrocytes after induction of neuroinflammation by systemic lipopolysaccharide (LPS) injection [85], a subtype identified by Liddelow et al. (2017) as neurotoxic A1 astrocytes [86]. Expression profiling of astrocytes induced by ischaemia revealed a different subtype of reactive astrocyte, which was termed the neuroprotective A2 astrocyte [86]. The minority of genes identified in both A1 and A2 astrocytes are termed pan-reactive genes, which include *Gfap*, *Vimentin (Vim)*, *Serpina3n*, and *Cxcl10*.

A more recent study comparing the transcriptional changes with age in three brain areas showed that the most highly up-regulated genes in all regions were reactive astrocyte-associated genes, mostly characteristic of the ‘A1 reactive astrocyte genes’ [79]. These genes included those involved in the complement (*C3* and *C4b*), antigen presentation (*H2-D1* and *H2-K1*), peptidase inhibitor (*Serpina3n*) and cytokine (*Cxcl10*) pathways. Nevertheless, a number of aged astrocytes also expressed a combination of A1 and A2 genes. A third study, although looking at four different brain regions and taking a different astrocyte isolation approach, also found similar genes upregulated with age, including pan-reactive gene *Serpina3n* and A1 genes *C3* and *C4b* [55], which are involved in synapse elimination [9, 91]. These last two studies both screened their DEGs for gene families involved with synapses and both found *Sparc* to be upregulated in aged astrocytes, which suggests an active role in decreasing synapse function [92]. Although a fourth study analysed astrocytes isolated from whole brain and of five ages ranging from young adult to middle-age, also *Serpina3n*, *C4b* and *Cxcl10* were significantly upregulated in astrocytes from the oldest compared to the youngest group [93]. Overall, aged murine astrocytes seem to become more reactive resembling the A1 phenotype. This reactive subtype is also present in different neurodegenerative diseases [94], however how expression of these reactivity-genes change with human ageing is largely unclear. One recent meta-analysis of human transcriptome data did analyse A1 and A2 signature genes, and though they found a reactive phenotype in aged astrocytes of the prefrontal cortex, A1 and A2 genes were equally over-represented [43]. Overall, it remains to be investigated how these genes behave throughout the ageing human brain and whether all astrocyte subpopulations respond similarly to neurotoxic stimuli. Moreover, the gene signature of ‘neurotoxic A1 astrocytes’ in humans may differ from those in mice, as it was recently shown that the transcriptional signatures of the human AD response in astrocytes was remarkably different from those observed in mice [95].

### Ageing-Induced Shift in Astrocyte Subpopulations

Astrocytes are a heterogeneous cell population and the composition shifts with ageing. The GFAP-high subpopulation as described above increases with age, at the expense of the homeostatic GFAP-low subpopulation [14]. Another subpopulation consists of the disease-associated astrocytes (DAAs). Albeit that these cells not only are present in disease, but also in the human brain during physiological ageing [14, 79]. DAAs show an upregulation of a subset of genes, including *GFAP*, *SERPINA3n*, and *VIM* [14]. This subset of upregulated genes comes together in processes such as endocytosis, complement cascade, development and differentiation, metabolic pathways, and inflammatory signalling. This DAA-gene-expression-pattern shows similarity to the previously in mice described A1 astrocytes [79]. The number of DAAs significantly increases in an early stage of AD and this shift in the astrocyte population composition is possibly a driving force of the switch between physiological to pathological ageing [14, 26]. This raises the question: What causes the shift in composition of astrocyte subpopulations? A possible candidate is the *APOE* gene, more precisely the E4 isoform which is the largest genetic risk factor for late-onset AD and mainly expressed in astrocytes [20, 22]. The effect of different APOE variants on astrocyte transcriptomics and its contribution to pathological ageing will be discussed in the next section.

## APOE4 in Human Astrocytes: Accelerating the Shift from Physiological to Pathological Ageing?

There are three different *APOE* alleles due to single nucleotide polymorphisms (SNPs) in the APOE gene: ε2, *ε3* and *ε4*. This results in three APOE protein isoforms with one or two differences in amino acids [96]. The *ε3* allele is the most common variant and 77% of the population has this allele. The *ε4* allele has a high prevalence in AD patients (40%) compared to the general population (15%) and is the main genetic risk factor for late-onset AD. These changes in the APOE4 protein lead to functional changes that are involved in the pathogenesis of AD [20, 97]. People with two *ε4* alleles have a 14.9 times higher chance of developing AD, show a decrease in AD age of onset, and present with a steeper progress in cognitive decline compared to people with two *ε3* alleles [20, 21].

APOE is a glycoprotein involved in the lipid metabolism of the brain. In homeostatic conditions, it is secreted predominantly by astrocytes and binds to lipids forming a lipoprotein particle. These particles bind on cell-surface APOE receptors (APOERs), primarily of the LDLR-family. For example, astrocyte-secreted APOE forms a lipoprotein with cholesterol, which binds to neuronal APOERs thereby supporting neuronal functioning [54]. The amount of APOE protein is SNP-dependent: ε2/ε2 results in the highest and *ε4*/*ε4* in the lowest levels of APOE in serum, CSF, and interstitial fluid (ISF) [24]. In human astrocytes specifically, it has been shown that *APOE* mRNA as well as intracellular and secreted levels of APOE protein are lower in *ε4*/*ε4* compared to *ε3*/*ε3* astrocytes [98, 99]*.* Also structurally, APOE3 and APOE4 differ significantly in isoform-dependent lipid-binding capacity, leading to altered lipoprotein sizes [100]. Besides the homeostatic functions, APOE is also involved in amyloid β (Aβ) clearance and the efficiency of clearance is isoform dependent [98, 101]. *APOE ε4* carriers show more accumulation of APOE and Aβ in plaques and clearance of Aβ is impaired [102, 103]. These differences are thought to underlie the effect of APOE4 on the increased risk to develop AD. However, it is not a direct effect of APOE4 on neurons, as astrocyte-conditioned medium containing APOE4 does not cause neuronal degeneration [104]. This suggests that, besides its role in Aβ clearance, other transcriptional and functional changes in astrocytes inflicted by *APOE* genotype may play a role in the increased risk to develop AD.

*APOE ε4* carriers without AD pathology already show differences in the brain compared to non-carriers. There are altered levels of complement proteins and other inflammatory markers in the CSF, such as reactive astrocyte markers (CCL4, S100β, YKL40, GFAP), compared to non-carriers of the same age [105]. Brain proteome analysis also shows that the GO term “regulation of inflammatory response” is positively correlated with *APOE ε4/ε4* genotype, while “synaptic transmission” and “post-synaptic membrane” are negatively correlated [20]. This is reflected in the inverse correlation of *APOE ε4* alleles with spine density in the dentate gyrus and hippocampal volume [106, 107]. *APOE ε4* carriers have an increased BBB breakdown in the hippocampus, which is also the brain region presenting age-related BBB permeability [108]. This is most likely mainly due to the effect of astrocyte-secreted APOE4 on pericytes and endothelial cells [109, 110].

Focussing on astrocytes specifically, in vitro studies show several differences between *APOE ε4/ε4* and *ε3/ε3* astrocytes (Fig. 1b). Regarding homeostatic functions, neuronal support and regulation of synaptic transmission are affected by *APOE* genotype. Cholesterol synthesis is the most significant positively enriched GO pathway in *APOE ε4/ε4* astrocytes and this is reflected in increased levels of intracellular and secreted cholesterol in these cells [98, 99]. This pathway is also enriched in the GFAP-high and DAA astrocyte subpopulations, which both increase in number with ageing [14]. Another characteristic of *APOE ε4/ε4* astrocytes is the upregulation of matrisome-related signals [99]. Matrisome is defined as a combination of ECM proteins (such as glycoproteins and collagens) and ECM associated factors (such as integrins, proteases, glypicans, plexins) [111]. The upregulation of molecules such as integrins also occurs in astrocytes in the aged human brain [25]. Glutamate receptor signalling is negatively correlated with age and the fact that the *APOE* genotype affects glutamate transporters and enzymes, corroborates the role of APOE4 in pathological ageing [25]. *APOE ε4*/*ε4* astrocytes show nuclear accumulation of excitatory amino acid transporter 1 (EAAT1) and cytoplasmic accumulation of GLUL [112]. Another homeostatic function related to the BDNF metabolism is regulated by APOE in a genotype-dependent way, as *APOE ε4*/*ε4* astrocytes have lower levels of intracellular BDNF than *ε3*/*ε3* astrocytes [113]. This suggests that an *APOE ε4*/*ε4* genotype amplifies the disruption of neuronal support and synaptic transmission functioning caused by ageing.

One study shows that astrocytes from an *APOE ε4*/*ε4* AD patient are more fibroblast-like compared to the arborized morphology of astrocytes of a control subject that is an *APOE ε3* carrier [112]. Besides, *APOE ε4*/*ε4* astrocytes show a general inflammatory profile, with high levels of secreted proteins such as SDF-1a (CXCL12), Gro-alpha/KC (CXCL1), MIP-1b (CCL4), Eotaxin (CCL11), IP-10 (CXCL10) and RANTES (CCL5), cytokines (IL-8, LIF and IL-6), and growth factors (VEGF-A, HGF and VEGF-D [99]. This corresponds to the higher levels of inflammatory markers in the CSF of *APOE ε4* carriers [105].

Next to these baseline differences between *APOE ε4*/*ε4* and *ε3*/*ε3* astrocytes, molecular differences are apparent in AD pathology. In Braak stage I-II subjects, the following groups of genes are downregulated in *APOE ε4* carriers: “cell signalling and communication”, “cytoskeleton”, “metabolism”, “DNA damage response”, and “transcription”. In contrast, “immune response” shows an upregulation in *APOE ε4*-carrying AD patients, on top of the consensus of reactive astrocytes in AD [30]. In vitro, *APOE ε4*/*ε4* astrocytes are less resistant to high levels of Tau leading to cell death, in comparison to *APOE ε3*/*ε3* astrocytes [114]. In addition to the age- and APOE-related changes that have been described so far, astrocytes dramatically change in AD. In the final section, we will review the age- and APOE4-related astrocyte changes in the context of AD for which both conditions are the largest risk factors.

## Linking Ageing, APOE4, and Alzheimer’s Disease in Human Astrocytes

Neuroinflammation is one of the major hallmarks of AD, in addition to Aβ plaques and neurofibrillary tangles [115]. Reactive astrocytes are already present in the prodromal phase of AD, indicated by high CSF levels of chitinase-3-like protein 1 (YKL-40), IL-15, intercellular adhesion molecule-1 (ICAM-1), and vascular cell adhesion protein 1 (VCAM-1) [116]. Also, hypertrophic astrocytes and increased expression of *GFAP*, *S100b*, *MAOB*, and *VIM* have been shown in AD patients [117–121]. Both aged astrocytes and APOE4 astrocytes show an inflammatory phenotype, which indicates that this is underlying the increased risk of developing AD with age and with *APOE ε4* genotype.

Glutamatergic neurotransmission is further affected when AD pathology becomes apparent. Expression of EAAT1 and EAAT2 is inversely related to AD pathology [30, 122, 123]. And in contrast to mGluR3, which is downregulated in ageing and AD, mGluR5 is upregulated in AD possibly due to effects of Aβ plaques via activation of NF-κB pathway [26, 124]. This change in mGluR expression increases calcium signalling in astrocytes, which is a generally observed phenotype in AD [30, 125, 126]. Regarding calcium signalling, rodent studies have shown that *APOE ε4*/*ε4* astrocytes have an increased calcium signalling which could mean that these astrocytes are less capable of compensating the ageing-induced changes thereby switching to pathological ageing/AD [127].

BBB integrity loss is something seen in both aged as well as *APOE ε4* carrying individuals. In AD patients, this phenotype is also present and the BBB integrity loss spreads to other brain regions rather than only hippocampus which is the case in ageing [72, 128, 129]. Importantly, BBB leakage is significantly correlated with cognitive decline [130]. This is (partly) caused by the downregulation of tight junction proteins in astrocytes, which are necessary for the integrity of the BBB [30]. AQP4, another protein involved in the BBB via perivascular endfeet, is increased in the hippocampus and temporal cortex of AD patients, but not the frontal cortex [42]. Reduction of AQP4 perivascular localization is associated with Aβ plaque density and cognitive decline [70]. In ageing, AQP4 is also upregulated, but not changed in localization indicating that mislocalization rather than upregulation is causative of pathological ageing.

*GJB6* is specifically upregulated in early AD pathology compared to controls without pathology, while *GJA1* is upregulated both in ageing and AD [26, 27]. However, Simpson et al. show significant transcriptional changes in astrocytes in the temporal cortex progressing from early to advanced stages of AD, amongst which a downregulation of the pathway “gap junctions” [30]. These contradicting studies have been performed in different stages of AD pathology indicating that the upregulation seen in ageing and early stages proceeds towards a downregulation in later stages of the disease [131].

## Discussion

In this review, we have described the molecular changes in astrocytes that occur with ageing in the human brain. However, there is only a limited number of studies investigating astrocyte-specific changes in the ageing human brain. Therefore, we not only described the enriched pathways, but also described specific proteins indicated to be changed in ageing by rodent studies and examined them in human studies. Although, rodent studies cannot be directly extrapolated to humans as the genetic variation is larger is human population. There are several limitations regarding the study of astrocytes in human brain development and ageing. There are roughly four sources of human astrocytes: fixed and fresh post-mortem brain tissue, surgical brain tissue, and in vitro human models. Fixed post-mortem brain tissue has allowed looking at cytoarchitecture of the human brain. However, this method only allows for static observations of astrocytes in a certain condition and mainly represents end-stage conditions of for example AD. Importantly, there is a limited availability of human post-mortem tissue of healthy young individuals making it difficult to make comparisons over time. Acute isolation of astrocytes from fresh post-mortem or surgical human brain tissue would overcome the first limitation and would allow for functional assays. However, it has been proven challenging to isolate and culture human post-mortem astrocytes [Hol & Middeldorp, unpublished data]. First of all, culture condition should be serum-free, as astrocytes are only exposed to serum in vivo in the case of BBB breakdown or injury and will therefore obtain a reactive phenotype when encountering serum [22]. Second, until the previous decade, a human astrocyte-specific surface marker was lacking to obtain a pure astrocyte culture [132]. In 2016, the Barres lab published a protocol to isolate human astrocytes with HepaCAM, a cell-adhesion glycoprotein, and culture them in a serum-free environment [22]. This method overcomes two of the limitations of fixed human post-mortem brain tissue, but the limited availability of human brain specimens makes this method not appropriate for extensive functional experiments.

With the development of a method to dedifferentiate human somatic cells into induced pluripotent stem cells (iPSCs), a whole new range of models has been established [133]. These human iPSCs can be differentiated into any cell type, among which astrocytes and neurons [134–137]. Human iPSC-derived astrocytes display key molecular and functional features of adult astrocytes, such as expression of GFAP, S100B, GLUL, CD44, and GJA1, calcium signalling, glutamate uptake, and support of synaptogenesis [134, 137]. In addition to differentiation into single cell types, iPSCs can also be differentiated into complex 3D neural tissues such as cerebral organoids [138, 139]. Cerebral organoids rely on intrinsic self-organizational capacity of cells to develop into several brain regions such as cerebral cortex and hippocampus [140]. These regions comprise numerous cell types, ranging from neurons, astrocytes, and oligodendrocytes, to microglia [139, 141, 142]. The 3D organization of these cell types together makes this model unique to study cell–cell interactions. Astrocytes can also be isolated from cerebral organoids based on CD49f expression to study/manipulate them in isolation [143]. CD49f is a laminin receptor expressed on iPSC-derived astrocytes and human foetal/mature astrocytes as well as human endothelial cells [22, 143]. Therefore, this marker might be suitable to isolate astrocytes from human brain tissue, in addition to HepaCAM, but this still needs confirmation.

These new models open up the possibility to study ageing and age-related neurodegenerative disease. This is approach is especially suitable for experiments into genetic components of ageing and age-related neurodegenerative diseases, such as APOE. In particular to study the effect of physiological ageing on *APOE ε4/ε4* astrocytes as there is essentially no healthy aged population with an *APOE ε4/ε4* genotype. For example, cerebral organoids from *APOE ε4* carrying AD patients show increased tau expression and phosphorylation [33]. However, it is unclear whether iPSC and iPSC-derived cells of aged individuals preserve ageing-associated characteristics in the epigenome [144]. This makes it more complicated to study physiological ageing in iPSC-derived models. To overcome this, ageing can be artificially induced in these models, by e.g. overexpression of progerin, a protein associated with premature ageing in progeria patients or by inducing oxidative stress which mimics ageing conditions [145, 146]. With these advancing techniques, more possibilities arise to study physiology and pathology of human astrocytes.

## Concluding Remarks and Future Perspective

Human astrocytes are an heterogenous population which are not solely defined by *GFAP* expression. Across subpopulations, aged human astrocytes are morphologically and transcriptionally different from young astrocytes implying age-related functional changes. Changes induced by ageing are similar to APOE4-induced changes in human astrocytes (Fig. 1), which could make the latter more vulnerable to age-related diseases such as AD. However, there are few studies into ageing of human astrocytes, mostly due to technical challenges and limited availability of tissue from young and old healthy individuals. State-of-the-art iPSC technology may help to fill the knowledge gap on transcriptional, morphological, and particularly functional changes on human ageing astrocytes.
